# ROBO1 p.E280* Loses the Inhibitory Effects on the Proliferation and Angiogenesis of Wild-Type ROBO1 in Cholangiocarcinoma by Interrupting SLIT2 Signal

**DOI:** 10.3389/fonc.2022.879963

**Published:** 2022-05-09

**Authors:** Tao Zhou, Yaodong Zhang, Yananlan Chen, Jijun Shan, Jifei Wang, Yirui Wang, Jiang Chang, Wangjie Jiang, Ruixiang Chen, Ziyi Wang, Xiaoli Shi, Yue Yu, Changxian Li, Xiangcheng Li

**Affiliations:** ^1^ Hepatobiliary Center, The First Affiliated Hospital of Nanjing Medical University, Nanjing, China; ^2^ Key Laboratory of Liver Transplantation, Chinese Academy of Medical Sciences, National Health Commission (NHC) Key Laboratory of Liver Transplantation, Nanjing, China

**Keywords:** cholangiocarcinoma, ROBO1, nonsense mutation, proliferation, angiogenesis

## Abstract

**Background:**

Cholangiocarcinoma (CCA) remains one of the most lethal malignancies with an increasing incidence globally. Through whole-exome sequencing of 67 CCA tissues, we identified new mutated genes in CCA, including MACF1, METTL14, ROBO1, and so on. The study was designed to explore the effects and mechanism of ROBO1 wild type (ROBO1^WT^) and ROBO1^E280*^ mutation on the progression of CCA.

**Methods:**

Whole-exome sequencing was performed to identify novel mutations in CCAs. *In vitro* and *in vivo* experiments were used to examine the function and mechanism of ROBO1^WT^ and ROBO1^E280*^ in cholangiocarcinoma. A tissue microarray including 190 CCA patients and subsequent analyses were performed to indicate the clinical significance of ROBO1.

**Results:**

Through whole-exome sequencing, we identified a novel CCA-related mutation, ROBO1^E280*^. ROBO1 was downregulated in CCA tissues, and the downregulation of ROBO1 was significantly correlated with poor prognosis. ROBO1^WT^ suppressed the proliferation and angiogenesis of CCA *in vitro* and *in vivo*, while ROBO1^E280*^ lost the inhibitory effects. Mechanically, ROBO1^E280*^ translocated from the cytomembrane to the cytoplasm and interrupted the interaction between SLIT2 and ROBO1. We identified OLFML3 as a potential target of ROBO1 by conducting RNA-Seq assays. OLFML3 expression was downregulated by ROBO1^WT^ and recovered by ROBO1^E280*^. Functionally, the silence of OLFML3 inhibited CCA proliferation and angiogenesis and was sufficient to repress the loss-of-function role of ROBO1^E280*^.

**Conclusions:**

These results suggest that ROBO1 may act as a tumor suppressor and potential prognostic marker for CCA. ROBO1^E280*^ mutation is a loss-of-function mutation, and it might serve as a candidate therapeutic target for CCA patients.

## Introduction

Cholangiocarcinoma (CCA) is a group of highly heterogeneous malignancies that originate from the biliary tree. CCA is classified as intrahepatic (iCCA), perihilar (pCCA), and distal CCA according to their anatomical sites (1). As the second most common primary liver malignancy, CCA constitutes about 15% of all primary liver tumors and 3% of all gastrointestinal tumors, and the incidence of CCA has been constantly increasing in the past few decades (2). Asymptomatic at an early stage and thus often diagnosed at an advanced stage, patients with CCA present a poor prognosis. Despite the advances in diagnosis and therapies, 5-year survival (7–20%) after resection has not changed significantly over time (1, 3, 4).

The development of next-generation sequencing technology has deepened our understanding of genomic alterations involved in the pathogenesis of CCA. Clinical trials testing special agents targeting hotspot IDH mutations and FGFR2 fusion mutation indicate that research on genomic alterations may have a clinical significance for the treatment of CCA (5, 6). Premature termination codons (PTCs) result in truncating mutations, including nonsense, frameshift, and splice-site mutations. Truncating mutations are often deleterious due to the generated truncated proteins being frequently non-functional or exerting dominant-negative effects. Approximately one-third of genetic diseases carry PTCs, and 10–30% of patients with inherited cancer carry nonsense mutations (7). More evidence shows that nonsense mutations play significant roles in tumorigenesis in several cancer types, including breast, lung, gastric, and pancreatic cancer and so on (8–11). In CCA, a nonsense mutation of p53 promoted cell proliferation and migration, and the nonsense mutation in LGR4 was associated with an increased risk of carcinogenesis (12, 13). Through whole-exome sequencing of 67 CCA tissues, we identified new mutated genes in CCA, including MACF1, METTL14, ROBO1, and so on. Our previous study demonstrated that METTL14-mediated m6A modification repressed the MACF1/β-catenin pathway in CCA, while METTL14^R298H^ mutation disrupted this mechanism. According to combined annotation–dependent depletion of the recurrent mutations, we also found that the p.E280* nonsense mutation of ROBO1 may be potentially deleterious in CCA.

ROBO was initially discovered in a *Drosophila* large-scale mutant screen for axonal guidance defects, and it was later identified as a cognate receptor for the secreted guidance molecule SLIT (14). The ROBO1 gene is located on chromosome 3 and encodes a transmembrane protein with a length of 1,551–1,655 aa, which is a member of the immunoglobin superfamily. ROBO1 contains five immunoglobin-like domains (Ig1–5) and three fibronectin type III modules (FNIII 1–3) in the extracellular region, one transmembrane region, and one intracellular region, including four conserved cytoplasmic domains (CC0–3). SLIT2 binds through its LRR2 domain to the Ig1 domain of ROBO1 (15). Studies have indicated that ROBO1 and its ligand SLIT2 play pivotal roles in neurodevelopment, and their deregulation is implicated in several neuropathological conditions. Besides this, the SLIT2/ROBO1 pathway seems to play contradictory roles in tumor progression. It was reported that ROBO1 was seen as an oncogene in the progression of cutaneous squamous cell carcinoma and mucoepidermoid carcinoma (16, 17). Nevertheless, an increasing number of studies indicated that the ROBO1 pathway may function as a tumor suppressor in a variety of malignancies. It was reported that the silence of the SLIT2/ROBO1 signaling pathway was involved in the progression of several malignancies, including gastric, pancreatic,and lung cancer (18–20). ROBO1 inhibited the cell proliferation of pancreatic cancer by regulating the CCNA2–CDK2 axis. In lung cancer, SLIT2/ROBO1 suppressed cancer cell migration through regulating the Myo9b/RhoA pathway. However, the function of ROBO1 and its mutations in CCA have rarely been reported.

In the present study, we aimed to explore the effects and mechanisms of ROBO1 and ROBO1^E280*^ mutation in CCA and their clinical value. We found that ROBO1 expression is downregulated in clinical CCA tissues, and the downregulation of ROBO1 is correlated with poor prognosis of CCA patients. *In vitro* and *in vivo* experiments revealed that ROBO1 suppresses the proliferation and angiogenesis of CCA, while E280* nonsense mutation of ROBO1 loses the tumor-suppressing effects through interruption of the SLIT2/ROBO1 signaling pathway. We also identified that OLFML3, as a potential target of ROBO1, promotes CCA progression, and the silence of OLFML3 is sufficient to repress the loss-of-function role of ROBO1^E280*^. Taken together, these results suggested that ROBO1^E280*^ is a loss-of-function mutation and represses the tumor suppressor function of ROBO1 in CCA.

## Materials and Methods

### Human Tissue Samples and Microarray

The tissue microarray was constructed by Outdo Biotech Company (Shanghai, China) from 190 CCA patients who underwent surgical procedures in 2006 to 2017 at The First Affiliated Hospital of Nanjing Medicine University. The expression of ROBO1 was evaluated by the grade semiquantitative scoring system. The intensity was classified as negative (0), weak (1), moderate (2), or strong (3), and the density of positive cells in the target region was scored as follows: 0–5% (0), 6–35% (1), 36–70% (2), and >70% (3). The overall score was calculated by multiplying intensity and density. Each microarray tissue point was scored by two independent pathologists, and the average score was taken as the final score. The score of tumor tissue/normal tissue >1 was defined as upregulated; otherwise, it was defined as downregulated.

The patients were followed up regularly until death or October 25, 2019. The use of clinical samples was approved by the Ethics Committee of The Affiliated Hospital of Nanjing Medical University. Written informed patient consent was obtained in accordance with regional regulations.

### Cell Culture

Human CCA cell lines RBE, HCCC9810, QBC939, and HuCCT1 and human umbilical vein endothelial cell line (HUVEC) were purchased from the Chinese National Human Genome Center (Shanghai, China). The cholangiocarcinoma cell lines were cultured in Dulbecco’s modified Eagle’s medium (Gibco, USA) supplemented with 10% fetal bovine serum (Gibco) and antibiotics (1% penicillin/streptomycin; Gibco). The HUVECs were cultured in F12-K medium (Gibco) containing 10% fetal bovine serum.

### Animal Experiment

Six-week-old male BALB/c nude mice (GemPharmatech Co., Ltd., China) were purchased to perform animal studies. Suspensions containing 1.0 × 10^7^ RBE cells were injected subcutaneously into the groin area of nude mice. The tumors were measured with a caliper every week until these were harvested 6 weeks later. Tumor volume is calculated according to the following formula: volume = width^2^ × length/2.

### HUVEC Tube Formation Assay

In total, 200 μl Matrigel (BD Bioscience, USA) was added to a 24-well plate and incubated at 37°C for 30 min. The HUVECs were resuspended with conditioned medium (CM) derived from tumor cells, and 1.0 × 10^5^ HUVECs were seeded into each well of the 24-well plate. Images were taken 8 h later.

### Chicken Chorioallantoic Membrane Assay

The chicken chorioallantoic membrane (CAM) assay was performed using specific pathogen-free fertilized chicken eggs (Jinan SAIS Poultry Co., Ltd, China). The eggs were incubated at 37°C under 80% humidity for 6 days. A 1-cm-diameter window was opened on the eggshell. The surface of the air sac floor was removed to expose the CAM. A 0.5-cm-diameter filter paper was placed onto the CAM, and 100 μl conditioned medium was added onto the filter paper before the window was closed with a sterile adhesive tape. After 3 days of incubation, the CAM was fixed with stationary solution (methanol/acetone = 1:1) and harvested. The photos of the CAMs were taken using a digital camera, and second- and third-order vessels were counted to assess the effects of the conditioned medium on angiogenesis.

### Lentivirus Infection and Sanger Sequencing

The ROBO1 wild type and c.838G>T (p.E280*) mutation overexpression lentiviruses were constructed by Genechem Co., Ltd. (Shanghai, China). The RBE and HCCC9810 cells were seeded in 6-well plates and then infected with lentivirus according to the manufacturer’s protocol. After 48 h, the medium was replaced with complete medium. The infected cells were treated with 10 μg/ml puromycin in order to select stable overexpressing cells. ROBO1 wild type and c.838G>T overexpression in RBE and HCCC9810, respectively, were confirmed by RT-qPCR and Sanger sequencing (Genewiz, China).

### RNA Sequencing

Total RNA was extracted from stable negative control, ROBO1^WT^, and ROBO1^E280*^ groups of RBE cells using TRIzol reagent (Invitrogen, California, USA) according to the manufacturer’s protocol. The concentration and the purity of total RNA were checked by NanoDrop 2000 Nucleic Acid and Protein Analyzer (Thermo Scientific, MA, USA). RNA-Seq analysis was performed by Shanghai Personalbio Technology Co., Ltd. (Shanghai, China).

### Statistical Analysis

All statistical analyses were performed with SPSS v24.0 (IBM, SPSS, Chicago, IL, USA) and Graphpad Prism 8 (GraphPad Software, La Jolla, CA, USA). Differences between the two groups were analyzed by Student’s *t*-test. Moreover, *χ* ^2^ test was used to analyze correlations between ROBO1 expression and clinicopathological variables. Overall survival (OS) and disease-free survival were analyzed with Kaplan–Meier methods, and log-rank test was applied for comparison. A multivariate analysis was performed using Cox proportional hazard regression model. Differences were considered as statistically significant when *p <0.05, **p < 0.01, or ***p <0.001. Supplementary information about materials and methods is provided in **Supplementary Material**.

## Results

### The ROBO1 ^E280*^ Mutation Was Found in CCA Patients

**Figure 1:**
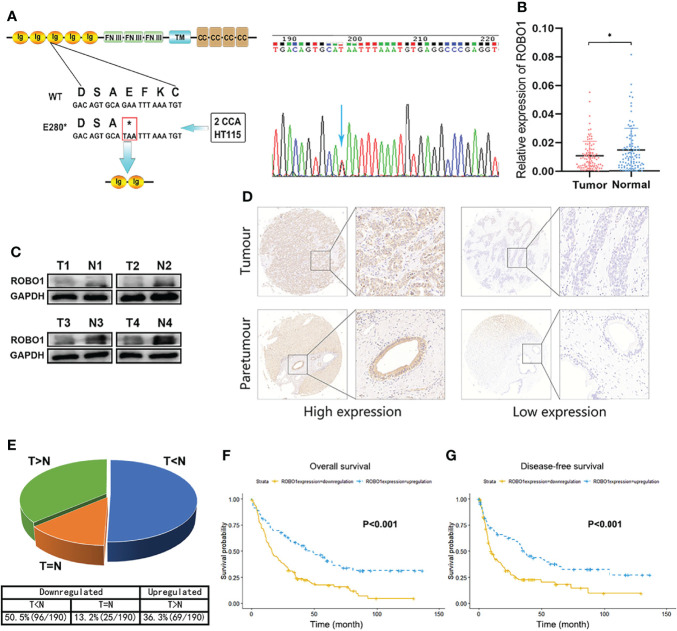
The downregulation of ROBO1 in cholangiocarcinoma (CCA) was associated with poor survival. **(A)** ROBO1 p.E280* mutation in CCA tissues. **(B)** RT-qPCR analysis of ROBO1 expression in CCA tissues and adjacent normal tissues. ROBO1 was downregulated in CCA tissues. **(C)** Western blotting analysis of typical ROBO1 expression in CCA tissues and adjacent normal tissues. **(D, E)** Immunohistochemistry of tissue microarrays showing the expression of ROB O1 as downregulated in CCA tissues. **(F, G)** The downregulation of ROBO1 in CCA was correlated with poor overall survival and disease-free survival after surgery. *P < 0.05.

To demonstrate the genomic landscapes of CCA, we performed whole-exome sequencing of 67 CCAs (including 43 pCCAs and 24 iCCAs). We identified significant mutated genes, including MACF1, METTL14, ROBO1, and so on. Furthermore, we performed combined annotation–dependent depletion (CADD) to evaluate the potential deleteriousness of gene mutations and listed them in descending order (**Supplementary Table S1**). ROBO1 p.E280* (c.838G>T, mutation rate 2.99%, found in 2 iCCAs of 67 CCAs) ranked first, with a CADD score of 38. The same mutation of ROBO1 also occurred in colorectal cancer cell line HT115 according to the COSMIC database. The ROBO1 p.E280* mutation induced a premature stop codon in the position of the 280th aa and may encode a truncated protein of ROBO1. The E280* mutant contains Ig1-2 domains and a part of Ig3 domain of the extracellular region, with the rest of the extracellular region and all the transmembrane and intracellular domains untranslated (**Figure 1A**). These observations indicated that the truncated mutations of ROBO1 may be highly deleterious in the progression of carcinogenesis, but the causality between this mutation and CCA has not yet been established.

### ROBO1 Is Downregulated in CCA Tissues and Associated With Poor Survival

Firstly, we explored the expression file of wild-type ROBO1 in CCA patients. The RT-qPCR of 99 pairs of CCA patients showed that ROBO1 mRNA expression was significantly downregulated in CCA tissues compared to their adjacent normal tissues (**Figure 1B**). Furthermore, the protein expression of ROBO1 was also significantly downregulated in CCA tissues (**Figure 1C**). To investigate the clinical significance, we analyzed the expression levels of ROBO1 by tissue microarray in 190 CCA patients. Representative immunohistochemical staining images of ROBO1 high/low expression patterns are shown in **Figure 1D**. The detailed immunohistochemistry (IHC) scores of the 190 pairs of tissues are shown in **Supplementary Figure S1**. As shown in **Figure 1E**, ROBO1 was downregulated in 63.7% (121 of 190) of CCAs compared with normal tissues.

The association between ROBO1 protein expression and the clinicopathological characteristics of CCA was analyzed and is shown in **Supplementary Table S2**. ROBO1 protein expression was significantly correlated with patient’s gender (*p* = 0.006) and marginally significantly correlated with serum CA199 (*p* = 0.087), tumor location (*p* = 0.068), T stage (*p* = 0.069), and clinical stage (*p* = 0.061). Besides these, the univariable analysis showed that ROBO1 expression, histological grade, tumor thrombus, nerve invasion, N stage, and clinical stage were significantly associated with the OS of CCA patients. Furthermore, the multivariate analysis showed that ROBO1 expression was an independent risk factor in patients with CCA (**Table 1**). The Kaplan–Meier survival curves demonstrated that CCA patients with upregulated ROBO1 had longer postoperative overall survival and disease-free survival than those with ROBO1 downregulated expression (**Figures 1F, G**). These results collectively suggested that the downregulation of ROBO1 may have a stimulatory role in the progression of CCA and predict poor survival.

**Table 1:** Univariate and multivariate analyses of the prognostic factors in Cholangiocarcinoma CCA patients.

Variable	Univariate analysis				Multivariate analysis		
	Cases	Events	*P*-value	χ2	HR	95% CI	*P*-value
Gender, male/female	118/72	93/51	0.174	1.850			
Age, ≤60/>60	98/92	72/72	0.226	1.466			
Diameter, <3/≥3	109/51	83/39	0.436	0.607			
Location, intrahepatic/perihilar	99/91	76/68	0.641	0.217			
Histological grade, I, I–II, II/II–III, III	89/90	63/72	**0.002** ^*^	9.903	1.837	1.274–2.648	**0.001** ^*^
Tumor thrombus, absent/present	154/30	114/24	**0.007** ^*^	7.249			0.200
Nerve invasion, absent/present	81/86	57/69	**0.022** ^*^	5.235			0.780
T stage, Tis–T1/T2–T4	69/117	49/92	0.054	3.706			
N stage, N0/N1, N2	138/49	96/46	**0.000** ^*^	12.666	1.585	1.070–2.349	**0.022** ^*^
M stage, M0/M1	185/1	140/1	0.539	0.377			
Clinical stage, I–II/III–IV	107/80	76/66	**0.004** ^*^	8.184			0.594
Surgical margin, R0/R1, R2	162/27	121/22	0.104	2.643			
ROBO1 expression, downregulated/upregulated	121/69	102/42	**0.000** ^*^	17.788	0.517	0.339–0.788	**0.002** ^*^

The bold values mean *P < 0.05 and the difference is significant.

### ROBO1^E280*^ Represses the Tumor-Suppressing Effects of ROBO1^WT^ on Proliferation in CCA Cells

The mRNA and protein expression levels of ROBO1 in CCA cell lines were detected by RT-qPCR and Western blot (**Supplementary Figures S2A, B**). RBE and HCCC9810 cells were used to detect the functional role of ROBO1^WT^ and ROBO1^E280*^ due to the relatively low expression and no congenital E280* mutation (**Supplementary Figure S2C**). Stably overexpressing ROBO1^WT^ and ROBO1^E280*^ cells were constructed with lentivirus transfection, and the transfection efficiency was confirmed by RT-qPCR and WB (**Supplementary Figures S2D, E**). As shown in **Supplementary Figure S2E**, ROBO1^E280*^ encoded a truncated protein (predicted molecular weight, 31 kDa; observed molecular weight, 43 kDa).

**Figure 2:**
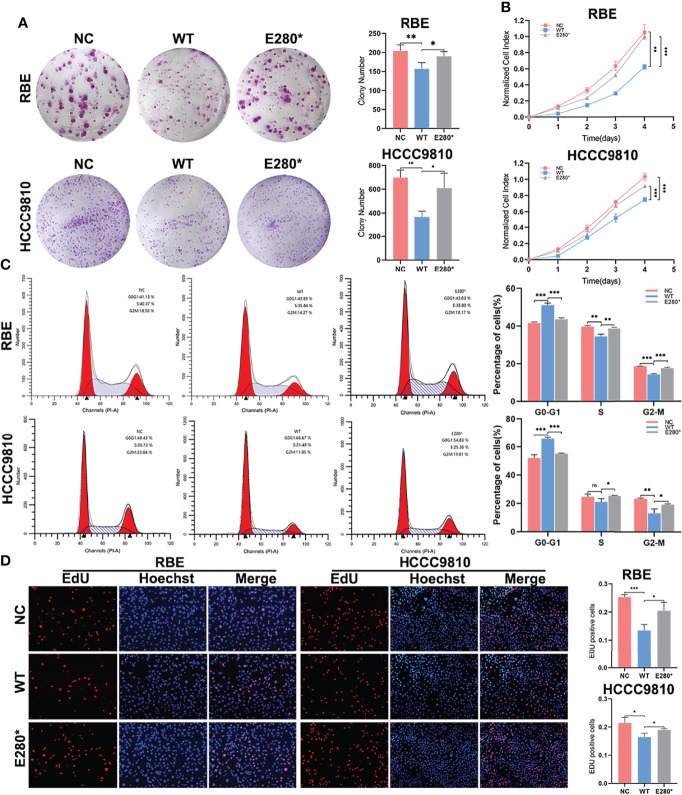
ROBO1^E280*^ repressed the tumor-suppressing effects of ROBO1^WT^ on proliferation in cholangiocarcinoma (CCA) cells. **(A)** The clone formation ability of CCA cells was impaired by ROBO1^WT^ and rescued by ROBO1^E280*^. **(B)** The overexpression of ROBO1^WT^ reduced the proliferation rate of CCA cells, which was reversed by the overexpression of ROBO1^E280*^. **(C)** Flow cytometry analysis of the cell cycle of CCA cells transfected with LV-ROBO1^WT^, LV-ROBO1^E280*^, and LV-NC. **(D)** ROBO1^E280*^ reversed the descending trend of EdU-positive cells in the ROBO1^WT^ group. *P < 0.05, **P < 0.01, ***P < 0.001.

**Figure 3:**
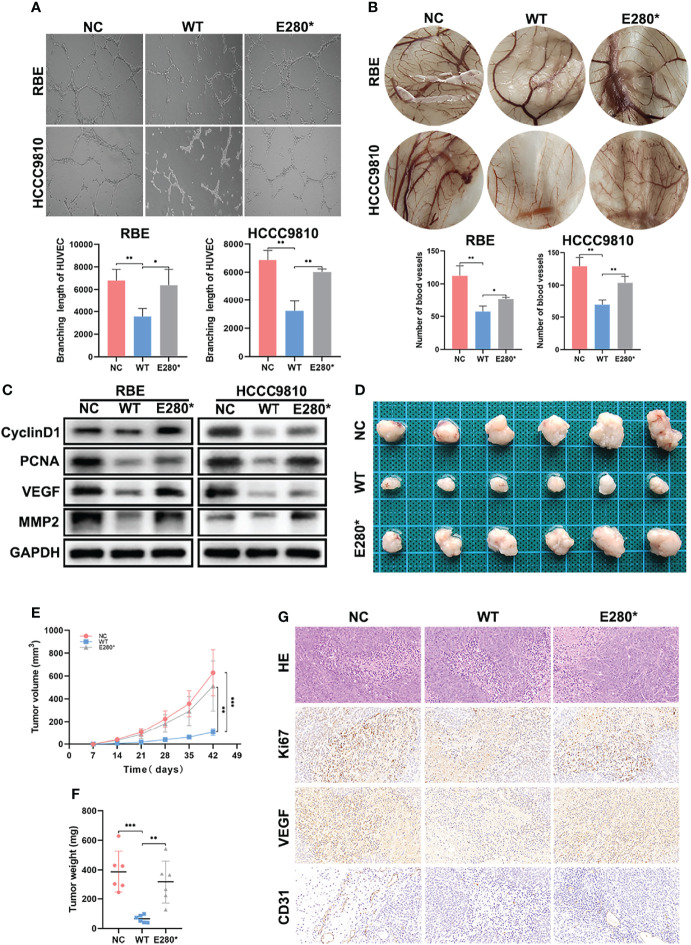
ROBO1^E280*^ reversed the inhibitory effects of ROBO1^WT^ on the angiogenesis and tumorigenesis of cholangiocarcinoma (CCA). **(A)** The branching length of HUVEC was significantly reduced by ROBO1^WT^ conditioned medium (CM) treatment and partially recovered by ROBO1^E280*^ CM treatment. **(B)** Chorioallantoic membrane assays showed a descending trend of vessel numbers in the ROBO1^WT^ group and a recovering trend in the ROBO1^E280*^ group. **(C)** Western blotting analysis showed a reverse trend of proliferative markers (cyclin D1 and PCNA) and angiogenic markers (VEGF and MMP2) in the ROBO1^E280*^ group compared with the ROBO1^WT^ group. **(D)** Xenograft tumors in nude mice generated with RBE cells transfected with LV-ROBO1^WT^, LV-ROBO1^E280*^, and LV-NC. **(E, F)** The growth of xenografts was suppressed in the ROBO1^WT^ group and rescued in the ROBO1^E280*^ group. **(G)** Ki67, EVGF, and CD31 expression in different groups of xenografts. *P < 0.05, **P < 0.01, ***P < 0.001.

To determine the role of wild-type ROBO1 and its E280* mutation in the proliferation of CCA cell lines, a clone formation assay showed that, compared to the NC group, the number of cell colonies decreased significantly in ROBO1-overexpressing cells, which was reversed by E280* mutation (**Figure 2A**). Consistently, in CCK8 assays, ROBO1 overexpression significantly decreased the absorbance at OD450, while cells with E280* mutations showed a significantly higher absorbance than wild-type ROBO1 (**Figure 2B**). In addition, flow cytometry assays showed that the percentage of cells in the G0–G1 phase was significantly higher in the ROBO1^WT^ overexpression group, while the percentage of cells in the S and G2–M phase decreased significantly. The E280* mutation reverses the phenotype of wild-type ROBO1 in the flow cytometry assays (**Figure 2C**, **Supplementary Figure S3**). The EdU assays also revealed that ROBO1 could inhibit the proliferation of CCA cells, while the mutant protein lost the function (**Figure 2D**). Furthermore, proliferation-related proteins cyclin D1 and PCNA were increased in ROBO1^E280*^ compared with ROBO1^WT^-overexpressing cells (**Figure 3C**). These data indicated that ROBO1^E280*^ represses the tumor-suppressing effects of ROBO1^WT^ on CCA cell proliferation.

### ROBO1^E280*^ Reverses the Inhibitory Effects of ROBO1^WT^ on the Angiogenesis and Tumorigenesis of CCA

Furthermore, we detected the effects of ROBO1^WT^ and ROBO1^E280*^ on tumor angiogenesis using HUVEC tube formation and CAM assays. As shown in **Figure 3A**, ROBO1^WT^ overexpression in CCA cells suppressed tube formation of HUVECs, while E280* lost the function of anti-angiogenesis. Similarly, the conditioned medium derived from ROBO1^WT^ cells inhibited angiogenesis in the CAM assays, while CM derived from ROBO1^E280*^ partially lost the anti-angiogenic effects (**Figure 3B**). In addition, the downregulation of angiogenesis-related proteins VEGF and MMP2 in ROBO1^WT^ cells was reversed by ROBO1^E280*^ (**Figure 3C**).

Subsequently, experiments *in vivo* were performed to evaluate the effects of ROBO1^WT^ and ROBO1^E280*^ on angiogenesis and tumorigenesis. The RBE cells stably transfected with LV-ROBO1^WT^, LV-ROBO1^E280*^, and LV-NC were injected subcutaneously into nude male mice. The results showed that ROBO1 overexpression significantly inhibited tumor growth *in vivo*, which was partially reversed by ROBO1^E280*^ (**Figures 3D**–**F**). IHC staining of xenografts showed that the expression of Ki67 and VEGF was decreased in the ROBO1^WT^ group, while it was recovered in the ROBO1^E280*^ group (**Figure 3G**). Besides this, higher microvascular density was observed in ROBO1^E280*^ compared with the ROBO1^WT^ group, using CD31 staining (**Figure 3G**). Taken together, these results indicated that ROBO1^E280*^ loses the inhibitory effects of ROBO1^WT^ on angiogenesis and tumorigenesis of CCA.

### ROBO1^E280*^ Interrupts SLIT2/ROBO1 Transmembrane Signal Transduction in CCA Cells

**Figure 4:**
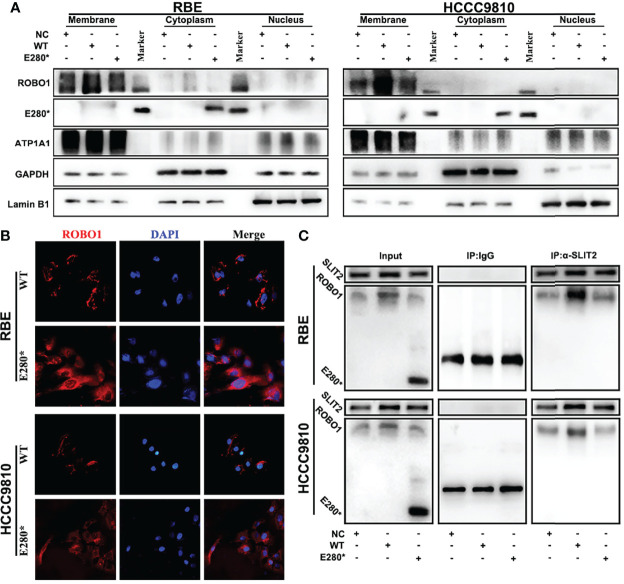
ROBO1^E280*^ interrupted SLIT2/ROBO1 transmembrane signal transduction in cholangiocarcinoma cells. **(A)** Subcellular fractionation analysis showed the membrane localization of ROBO1^WT^ and the cytoplasmic localization of ROBO1^E280*^ in RBE cells. **(B)** Immunofluorescence staining revealed the subcellular localization of ROBO1^WT^ and ROBO1^E280*^ in RBE cells. **(C)** Co-immunoprecipitation, performed using either control IgG or anti-SLIT2 antibodies, showed that the ROBO1^E280*^ was not detected in the immunoprecipitates of the anti-SLIT2 antibody.

To explore the mechanism under the loss-of-function phenotype of ROBO1^E280*^, we performed subcellular fractionation analysis to identify the subcellular localization of the truncated protein. Western blotting analysis revealed that ROBO1^WT^ was detected on the cytomembrane, while ROBO1^E280*^ was found in the cytoplasm (**Figure 4A**). Immunofluorescence staining further confirmed the translocation of ROBO1^E280*^ (**Figure 4B**). Given that SLIT2 ligands interact with ROBO1 receptors on the cytomembrane, we thus performed co-immunoprecipitation assays to examine whether the SLIT2/ROBO1 interaction was affected by E280* mutation. The anti-SLIT2 antibody specifically co-immunoprecipitated ROBO1^WT^, while ROBO^E280*^ was not detected in the immunoprecipitates of cells overexpressing ROBO1^E280*^ (**Figure 4C**), which revealed that ROBO1^E280*^ failed to interact with SLIT2. According to these results, the SLIT2/ROBO1 transmembrane signal transduction is interrupted by the E280* mutation of ROBO1.

### ROBO1^E280*^ Disrupts SLIT2-Induced Tumor-Suppressing Effects on Proliferation and Angiogenesis in CCA

**Figure 5:**
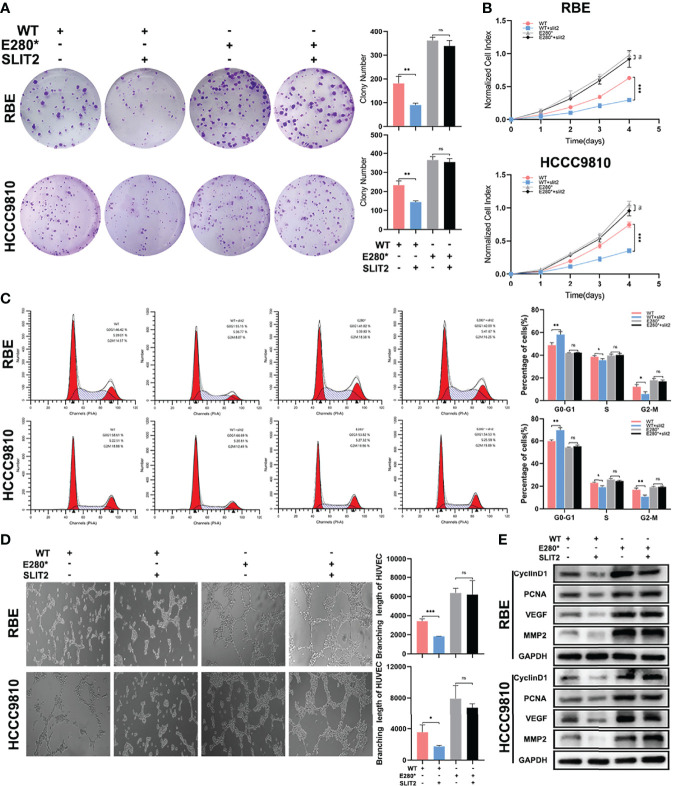
ROBO1^E280*^ disrupted the SLIT2-induced tumor-suppressing effects on proliferation and angiogenesis in cholangiocarcinoma. **(A)** SLIT2 treatment impaired the clone formation ability of ROBO1^WT^ cells but not that of ROBO1^E280*^ cells. **(B)** SLIT2 treatment reduced the proliferation rate of ROBO1^WT^ cells but not that of ROBO1^E280*^ cells. **(C)** Flow cytometry analysis of the cell cycle of wild-type and mutated RBE cells treated by SLIT2. **(D)** SLIT2 treatment did not decrease the branching length of human umbilical vein endothelial cell line in the ROBO1^E280*^ group as in the ROBO1^WT^ group. **(E)** Western blotting analysis of the expression trend of cyclin D1, PCNA, VEGF, and MMP2 after SLIT treatment. *P < 0.05; **P < 0.01; ***P < 0.001; ns, *P* > 0.05.

Furthermore, we performed functional experiments to verify the interruption of SLIT2/ROBO1 signal by E280* mutation of ROBO1. As shown in **Figure 5A**, SLIT2 treatment significantly decreased the number of colonies in ROBO1^WT^ cells, while no significant decrease was observed in ROBO1^E280*^ cells. Consistent with the clone formation assays, the absorbance at OD450 was significantly decreased with SLIT2 treatment in ROBO1^WT^ cells, while SLIT2 treatment in ROBO1^E280*^ cells showed no significant differences (**Figure 5B**). The flow cytometry cell cycle analyses and EdU assays demonstrated similar results (**Figure 5C** and **Supplementary Figures S4A, B**). Furthermore, SLIT2-treated ROBO1^WT^ CCA cells significantly decreased HUVEC tube formation compared with untreated cells, which is not observed in ROBO1^E280*^ cells (**Figure 5D**). Consistent with the functional experiments, western blotting analysis showed that cyclin D1, PCNA, VEGF, and MMP2 were significantly downregulated in SLIT2-treated ROBO1^WT^ cells, which was not observed in SLIT2-treated ROBO1^E280*^ cells (**Figure 5E**). These results revealed that ROBO1^E280*^ disrupts the SLIT2-induced tumor-suppressing effects on proliferation and angiogenesis in CCA.

### OLFML3 Is a Potential Target Regulated by ROBO1^WT^ and ROBO1^E280*^

**Figure 6:**
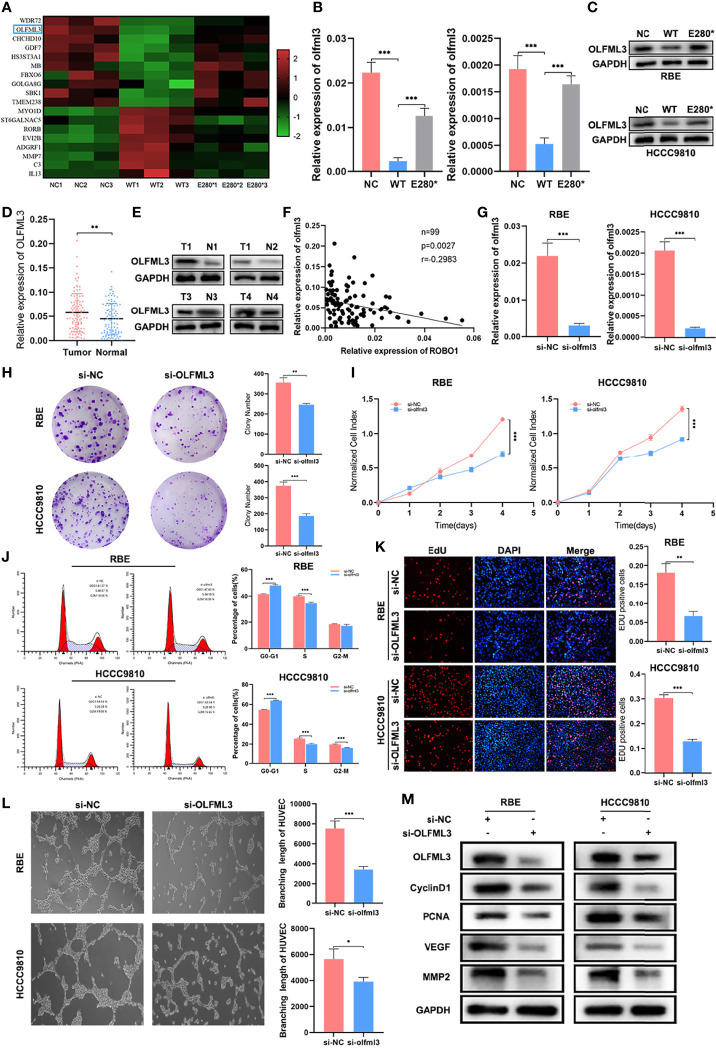
OLFML3 was a potential target regulated by ROBO1^WT^ and ROBO1^E280*^. **(A)** Heat map of differentially expressed genes that have a reverse expression trend in the ROBO1^E280*^ group compared with the ROBO1^WT^ group. **(B, C)** Verification of OLFML3 expression in RBE and HCCC9810 cell line by RT-qPCR and western blotting analyses. OLFML3 expression was downregulated by ROBO1^WT^ and recovered by ROBO1^E280*^. **(D)** The RT-qPCR analysis of OLFML3 expression showed an upregulated trend in cholangiocarcinoma (CCA) tissues. **(E)** Western blotting analysis of typical OLFML3 expression in CCA and normal tissues. **(F)** Scatter diagram illustrating the negative correlation between ROBO1 and OLFML3 mRNA expression. **(G)** Knockdown of OLFML3 in RBE and HCCC9810 cell line. **(H)** The knockdown of OLFML3 significantly impaired the clone formation ability of RBE and HCCC9810 cells. **(I)** The knockdown of OLFML3 significantly reduced the proliferation rate of RBE and HCCC9810 cells. **(J)** Flow cytometry analysis of the cell cycle of RBE and HCCC9810 cells transfected with si-OLFML3 and si-NC. **(K)** The EdU-positive RBE and HCCC9810 cells were decreased by the knockdown of OLFML3. **(L)** The human umbilical vein endothelial cell line branching length was significantly decreased by the knockdown of OLFML3 in RBE and HCCC9810 cells. **(M)** Western blotting analysis showed a lower expression of cyclin D1, PCNA, VEGF, and MMP2 in si-OLFML3 cells. *P < 0.05, **P < 0.01, ***P < 0.001.

To explore the potential target of ROBO1^WT^ and ROBO1^E280*^, we conducted RNA-seq in negative control, ROBO1^WT^, and ROBO1^E280*^ cells. We screened a set of target genes whose expression was reversed in ROBO1^E280*^ cells compared with ROBO1^WT^ cells (**Figure 6A**). Among these genes, RT-qPCR and western blotting analysis verified that OLFML3, which plays a role in tumor-related angiogenesis (21, 22), was decreased in the ROBO1^WT^ group, while it was recovered in the ROBO1^E280*^ group (**Figures 6B, C**). Subsequently, we detected OLFML3 mRNA expression in clinical samples. The RT-qPCR of 99 pairs of CCA tissues demonstrated that OLFML3 mRNA expression was significantly upregulated in CCA tissues compared with normal tissues (**Figure 6D**). **Figure 6E** showed the typical OLFML3 protein expression in CCA tissues and matched normal tissues. Moreover, linear regression analysis revealed that OLFML3 mRNA expression was significantly negatively correlated with ROBO1 expression (**Figure 6F**).

Next, we performed a series of functional experiments to elucidate the role of OLFML3 in the proliferation and angiogenesis of CCA. The knockdown efficiency of OLFML3 siRNA was verified using RT-qPCR and western blotting analysis (**Figures 6G, M**). As shown in **Figure 6H**, the knockdown of OLFML3 significantly decreased the colony numbers compared with the control cells. In CCK8 assays, the absorbance of OD450 was also decreased in OLFML3-knockdown cells (**Figure 6I**). In addition, the percentage of G0–G1 phase cells increased, while that of S phase cells decreased in OLFML3-knockdown cells compared with control cells. In HCCC9810 OLFML3-knockdown cells, the percentage of G2–M cells also showed a significant decrease (**Figure 6J** and **Supplementary Figure S5A**). Furthermore, the EdU assays showed that the percentage of EdU-positive cells was reduced in OLFML3-knockdown cells compared with the control cells (**Figure 6K**). Subsequently, tube formation assays demonstrated that the depletion of OLFML3 reduced the angiogenic capacity of HUVECs (**Figure 6L**). Accordingly, western blotting analysis revealed that the expression of cyclin D1, PCNA, VEGF, and MMP2 was downregulated by the knockdown of OLFML3 (**Figure 6M**). These results indicated that OLFML3 may be a target gene of ROBO1 and act as a potential oncogene of CCA.

### OLFML3 Is Essential for ROBO1^E280*^-Induced Proliferation and Angiogenesis in CCA

**Figure 7:**
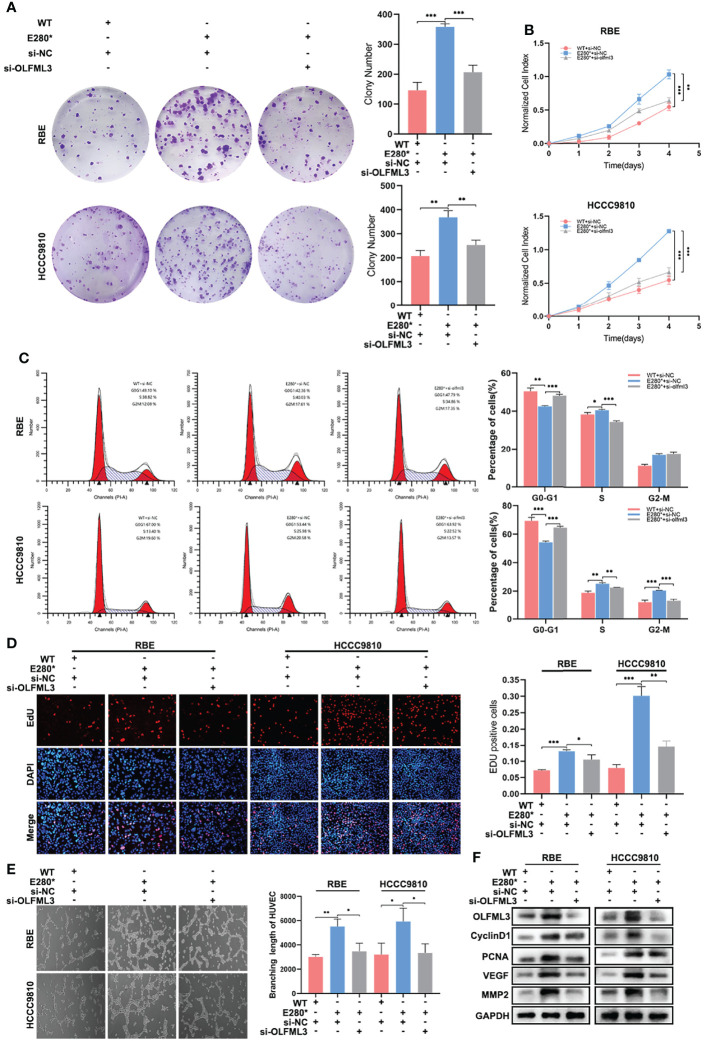
OLFML3 was essential for ROBO1^E280*^-induced proliferation and angiogenesis in cholangiocarcinoma. **(A)** The knockdown of OLFML3 impaired the clone formation ability of ROBO1^E280*^ cells. **(B)** The knockdown of OLFML3 reduced the proliferation rate of ROBO1^E280*^ cells. **(C)** Flow cytometry analysis of the cell cycle of ROBO1^WT^ cells transfected with si-NC, ROBO1^E280*^ cells transfected with si-NC, and of ROBO1^E280*^ cells transfected with si-OLFML3. **(D)** The ascending proportion of EdU-positive cells in the ROBO1^E280*^ group was rescued by the knockdown of OLFML3. **(E)** The knockdown of OLFML3 decreased the branching length of human umbilical vein endothelial cell line in the ROBO1^E280*^ cells. **(F)** Western blotting analysis of proliferative markers (cyclin D1 and PCNA) and angiogenic markers (VEGF and MMP2). *P < 0.05, **P < 0.01, ***P < 0.001.

Subsequently, we knocked down OLFML3 in ROBO1^E280*^ cells and examined whether the knockdown of OLFML3 could restore the lost tumor-suppressing effects of ROBO1^E280*^. In clone formation assays, the knockdown of OLFML3 decreased the increased colony number in ROBO1^E280*^ cells (**Figure 7A**). Similar results were observed in CCK8 assays (**Figure 7B**). In cell cycle assays, the depletion of OLFML3 in ROBO1^E280*^ elevated the proportion of cells in G0–G1 phase and decreased that of cells in S phase (**Figure 7C** and **Supplementary Figure S6A**). Besides this, the EdU assays showed that the depletion of OLFML3 partly decreased the EdU-positive proportion in ROBO1^E280*^ cells (**Figure 7D**). Moreover, the enhanced angiogenic capacity of ROBO1^E280*^ was reversed by the depletion of OLFML3 (**Figure 7E**). Western blotting analysis showed that the increased expressions of cyclin D1, PCNA, VEGF, and MMP2 in ROBO1^E280*^ cells were reversed by the knockdown of OLFML3 (**Figure 7F**). Taken together, the above-mentioned results revealed that OLFML3 is essential for ROBO1^E280*^-induced proliferation and angiogenesis in CCA.

## Discussion

Previous studies have found several aberrations in ROBO1 that may be linked to the initiation and development of malignant tumors, although these variants have not yet been verified for causal implications (23, 24). Our whole-exome sequencing results identified new significant mutated genes, such as ROBO1. ROBO1^E280*^ nonsense mutation induced a premature stop codon and encoded a truncated protein of ROBO1. In this project, we first reported that ROBO1 was downregulated in CCA tissues, and the downregulation of ROBO1 was associated with poor survival of CCA patients. *In vitro* and *in vivo* functional studies demonstrated that the overexpression of ROBO1^WT^ suppressed the proliferation and angiogenesis of CCA, while the overexpression of ROBO1^E280*^ lost the tumor-suppressing effects of ROBO1^WT^. Subcellular fractionation analysis and immunofluorescence staining revealed the cytoplasmic localization of the truncated protein encoded by ROBO1^E280*^, which was different from the membrane localization of the full-length protein encoded by ROBO1^WT^. Taken together, these results indicated that ROBO1 may serve as a tumor suppressor in CCA progression, and ROBO1^E280*^ is a loss-of-function mutation, interrupting the SLIT2/ROBO1 signaling pathway and reversing the tumor-suppressing effects of wild-type ROBO1.

Angiogenesis is described as the formation of new blood vessels by the expansion of the surrounding vascular network, which is regulated by various pro- and anti-angiogenic factors (25). Several drugs targeting these factors, like VEGF, have shown a statistically significant survival improvement in clinical trials (26–28). Currently, there still exist controversies concerning the function of ROBO1 in angiogenesis. Several studies have shown that ROBO1 has important functions in angiogenesis. The activation of ROBO1 in vascular endothelial cells promoted angiogenesis (29). Furthermore, in human renal glomerular endothelial cells, the activation of SLIT2/ROBO1 signaling may contribute to angiogenesis through the PI3K/Akt/VEGF pathway (30). On the contrary, several studies have shown that the SLIT2/ROBO1 signal suppresses neovascular formation, especially in tumor angiogenesis. In breast cancer cells, the activation of ROBO1 signaling restrains tumorigenesis by blocking the PI3K/Akt/β-catenin pathway (31). Furthermore, ROBO4 stabilizes the vascular network by inhibiting pathologic angiogenesis and endothelial hyperpermeability (32). Tavora et al. reported that the activation of SLIT2 in the endothelium facilitated metastasis, while tumoral SLIT2 repressed metastasis (33), indicating that a single gene may have opposite effects on tumor progression depending on its different cellular source. Our results demonstrated that the overexpression of ROBO1^WT^ suppressed the angiogenesis and tumorigenesis of CCA *in vitro* and *in vivo*, while the overexpression of ROBO1^E280*^ reversed the tumor-suppressing effects of ROBO1^WT^. These results indicated that ROBO1 exerts an inhibitory role in CCA through regulating angiogenesis and that ROBO1^E280*^ reverses the tumor-suppressing effect on angiogenesis.

Through RNA sequencing, we screened differentially expressed genes and found that OLFML3 expression was downregulated by ROBO1^WT^ and recovered by ROBO1^E280*^. OLFML3, a member of the olfactomedin domain-containing secreted glycoprotein family, was reported to be involved in neovascular formation during embryonic development and tumor progression (21, 22, 34, 35). Miljkovic et al. reported that targeting OLFML3 suppressed tumor growth *via* impairing angiogenesis and pericyte coverage (35). Furthermore, Stalin et al. reported that targeting OLFML3 increased the efficiency of anti-PD1-based immunotherapy (22). These studies indicated that OLFML3 may act as a potential therapeutic target towards tumor angiogenesis. In our study, the results showed that the expression of OLFML3 was negatively correlated with ROBO1 expression in CCA tissues and that the negative regulation of OLFML3 by ROBO1^WT^ was reversed by ROBO1^E280*^. We found that the knockdown of OLFML3 inhibited the proliferation and angiogenesis of CCA cells. Furthermore, the lost inhibitory function on proliferation and angiogenesis of ROBO1^E280*^ can be recovered by the knockdown of OLFML3. These results indicated that OLFML3 may be a target gene of ROBO1 and may serve as a potential therapeutic target for CCA patients with ROBO1^E280*^. However, the direct interaction and its mechanism between ROBO1 and OLFML3 need further investigation.

In the study, we confirmed ROBO1 as a tumor suppressor in CCA patients and that the downregulation of ROBO1 is correlated with poor prognosis. Additionally, we identified a deleterious nonsense mutation, ROBO1^E280*^, in CCA, which loses the tumor-suppressing function of wild-type ROBO1 due to its translocation and interruption of the SLIT2/ROBO1 signal pathway. Furthermore, OLFML3 is a potential target gene of ROBO1, and its inhibition restores the lost tumor-suppressing effects of ROBO1^E280*^. Taken together, these results suggested that ROBO1 may act as a tumor suppressor and potential prognostic marker for CCA. ROBO1^E280*^ mutation is a loss-of-function mutation, and it may serve as a candidate therapeutic target for CCA patients.

## Data Availability Statement

The original contributions presented in the study are included in the article/**Supplementary Materials**, further inquiries can be directed to the corresponding authors.

## Ethics Statement

The studies involving human participants were reviewed and approved by the Ethics Committee of The First Affiliated Hospital of Nanjing Medical University. The patients/participants provided their written informed consent to participate in this study.

## Author Contributions

TZ, YZ, XL, CL, and YY designed the study. TZ, YC, JS, JW, YW, JC, WJ, RC, ZW, and XS collated the data, carried out data analyses, and produced the initial draft of the manuscript. TZ, YC, JS, and JW conducted experiments. XL, CL, and YY supervised the research and reviewed the manuscript. All authors contributed to the article and approved the submitted version.

## Funding

This study was supported by the Key Research and Development Program of Jiangsu Province (BE2016789).

## Conflict of Interest

The authors declare that the research was conducted in the absence of any commercial or financial relationships that could be construed as a potential conflict of interest.

## Publisher’s Note

All claims expressed in this article are solely those of the authors and do not necessarily represent those of their affiliated organizations, or those of the publisher, the editors and the reviewers. Any product that may be evaluated in this article, or claim that may be made by its manufacturer, is not guaranteed or endorsed by the publisher.
