# Supplementary material for: ROBO1 p.E280* Loses the Inhibitory Effects on the Proliferation and Angiogenesis of Wild-Type ROBO1 in Cholangiocarcinoma by Interrupting SLIT2 Signal
Source: Front Oncol. 2022 May 9;12:879963. doi: 10.3389/fonc.2022.879963 (PMC9124974; doi:10.3389/fonc.2022.879963)
Supplement: Supplementary file 2 [file DataSheet_2.pdf]

Supplementary Table. 2 Association of ROBO1 expression with clinicopathological features of CCA.

Clinicopathological features	ROBO1 expression downregulated	ROBO1 expression upregulated	P value	χ2
**All cases**	121(63.7%)	69(36.3%)		
**Gender**			**0.006**	**7.578**
Male	84(69.4%)	34(49.3%)		
Female	37(30.6%)	35(50.7%)		
**Age**			0.901	0.015
≤60	62(51.2%)	36(52.2%)		
＞60	59(48.8%)	33(47.8%)		
**CA199(U/L)**			0.087	2.929
≤37	11(9.1%)	10(14.5%)		
＞37	52(43.0%)	20(29.0%)		
**CEA(ng/ml)**			0.566	0.330
≤5	41(33.9%)	18(26.1%)		
＞5	21(17.4%)	12(17.4%)		
**AFP(ng/ml)**			0.746	0.105
≤20	69(57.0%)	34(49.3%)		
＞20	3(2.5%)	2(2.9%)		
**Diameter(cm)**			0.316	1.006
≤5	73(60.3%)	36(52.2%)		
＞5	30(24.8%)	21(30.4%)		
**Tumor No.**			0.578	0.310
1	64(52.9%)	34(49.3%)		
≥2	6(5.0%)	2(2.9%)		
**Location**			0.068	3.335
Intrahepatic	57(47.1%)	42(60.9%)		
Perihilar	64(52.9%)	27(39.1%)		
**Histological grade**			0.195	1.681
I/I-II/II	52(43.0%)	37(53.6%)		
II-III/III	61(50.4%)	29(42.0%)		
**Nerve invasion**			0.273	1.201
Absent	49(40.5%)	32(46.4%)		
Present	59(48.8%)	27(39.1%)		
**Tumor thrombus**			0.653	0.202
Absent	96(79.3%)	58(84.1%)		
Present	20(16.5%)	10(8.3%)		
**T stage**			0.069	3.312
Tis-T1	38(31.4%)	31(44.9%)		
T2-T4	80(66.1%)	37(53.6%)		
**N stage**			0.187	1.742
N0	84(69.4%)	54(78.3%)		
N1, N2	35(28.9%)	14(20.3%)		
**M stage**			0.447	0.579
M0	117(96.7%)	68(98.6%)		
M1	1(0.8%)	0(0.0%)		
**Clinical stage**			0.061	3.502
I/II	62(51.2%)	45(65.2%)		
III/IV	57(47.1%)	23(33.3%)		
**Surgical margin**			0.711	0.137
R0	102(84.3%)	60(87.0%)		
R1，R2	18(14.9%)	9(13.0%)		
