# Supplementary material for: ROBO1 p.E280* Loses the Inhibitory Effects on the Proliferation and Angiogenesis of Wild-Type ROBO1 in Cholangiocarcinoma by Interrupting SLIT2 Signal
Source: Front Oncol. 2022 May 9;12:879963. doi: 10.3389/fonc.2022.879963 (PMC9124974; doi:10.3389/fonc.2022.879963)
Supplement: Supplementary file 3 [file DataSheet_3.docx]

Supplementary Material

# Supplementary materials and methods

## CCK8 assay

CCK8 assay kit (Dojindo, Japan) was used to assess cell proliferation. Cells were seeded in 96-well plates at 1×10^3^ cells/well. In each day of the subsequent 5 days, 100μ medium containing 10μ CCK8 reagent was added into each well. The absorbance at 450nm was measured with a microplate reader.

## Clone formation assay

Cells were seeded in 6-well plates at 1×10^3^/well and incubated at 37°C for 2 weeks. The cells were fixed with 4% paraformaldehyde (Beyotime, China) for 15 min and stained with 0.1% crystal violet (Beyotime, China) for 30min. The colonies were counted and photographed.

## Cell cycle analysis

Flow cytometry was used to analyze the cell cycle using cell cycle staining kit (MultiSciences, China). After 48h of transfection, cells were harvested and washed with PBS and then fixed in 75% ethanol overnight at -20°C. Subsequently, cells were washed with PBS three times and incubated with propidium iodide (10mg/ml) for 30min at room temperature in the shadow. The percentage of cells in each phase was analyzed with FACS Calibur flow cytometer, Cell Quest (BD Biosciences, USA).

## Edu assay

EdU cell proliferation kit (Beyotime, China) was used for EdU assay to assess cell proliferation. Cells were planted in 24-well plate at 80% confluence and cultured for 24h, followed by 2h incubation with 10μM EdU medium. The cells were fixed in 4% paraformaldehyde for 15min and permeabilized with 0.3% Triton for 15 min. Sequentially the cells were stained with Alexa Fluor 555 azide for 30 min and Hoechst 33342 for 10 min in the dark. The cells were photographed under a fluorescence microscope (Olympus, Japan).

## RNA extraction and RT-qPCR

Total RNA was extracted from cell lines and tissue samples using TRIzol reagent (Invitrogen, USA) according to the manufacturer’s protocol. Reverse transcription was performed using HiScript Q RT SuperMix (Vazyme, China). RT-qPCR was performed with AceQ qPCR SYBR Green Master Mix (Vazyme, China). RNA relative expression was calculated using 2^-△△CT^ method with GAPDH as an endogenous control. The sequences of the related primers were listed below.

Primers for ROBO1:

Forward 5’-CGCCCCACACCCACTATTG-3’

Reverse 5’-GAAGTCATCCCGAAGTATGGC-3’

Primers for OLFML3:

Forward 5’-TCCTTTTGTCATGGTCGGGAC-3’

Reverse 5’-TAAAGCAGCTAGTCGGCGTTC-3’

Primers for GAPDH:

Forward 5’- GGAGCGAGATCCCTCCAAAAT -3’

Reverse 5’- GGCTGTTGTCATACTTCTCATGG -3’

## Total protein extraction, subcellular protein fractionation, and western blot analysis

Total protein was extracted from cell lines with NP-40 Lysis Buffer (Beyotime, China) supplemented with 1mM PMSF. Cell Fractionation Kit (Cell Signal Technology, USA) was purchased to fractionate proteins into membranous, cytoplasmic and nuclear fractions according to the manufacturer’s protocol. The proteins were separated using SDS-PAGE electrophoresis and transferred to PVDF membranes. The membranes were blocked in 5% nonfat powered milk TBST solution for 2 hours and incubated overnight in appropriate primary antibody at 4°C. The membranes were incubated in the corresponding HRP secondary antibody for 2 hours at room temperature. The related antibodies were listed at the end of this file.

## Immunohistochemical staining and immunofluorescence staining

Microarrays of cholangiocarcinoma tissues and sections of mice xenografts were prepared for immunohistochemical staining. The slides were immersed in 3% H2O2 for 5 min at room temperature to block endogenous peroxidase activity and incubated in sodium citrate buffer for 15 min at 95°C for antigen retrieval. After being blocked with 5% normal goat serum for 10 min, the slides were incubated with corresponding antibodies overnight at 4°C, followed by incubation with appropriate secondary antibody for 1 hour at room temperature. Nuclei were visualized by DAPI (Beyotime, China) staining. The images were taken through a fluorescence microscope (Olympus, Japan).

For immunofluorescence, cells attached to slides were fixed with 4% paraformaldehyde and permeabilized with Immunostaining Permeabilization Buffer with Saponin (Beyotime, China). After washing, the sliders were blocked with 5% BSA in PBS for 1 hour at room temperature and then incubated with appropriate primary antibody overnight at 4°C. The cells were washed three times and incubated with corresponding secondary antibody for 1 hour at room temperature. Nuclei were stained with DAPI. The slides were photographed under a fluorescence microscope (Olympus, Japan).

## Immunoprecipitation (IP)

Cells were treated with conditioned medium containing SLIT2 recombinant protein (6nM, Abcam) for 24h to make SLIT2 protein highly enriched on membrane. Cells were harvested using NP-40 lysis buffer and placed on ice for 30 min, sonicated and subsequently centrifuged at 15000 rpm for 15 min at 4°C. 1 μ normal rabbit IgG and 20 μ protein A agarose beads (Cell Signal Technology, USA) were added to 500 μ collected supernatant，shaken on a horizontal shaker for 1 hour in order to eliminate non-specific binding proteins. Centrifuged at 2500 rpm for 10 min, the supernatant was transferred to a new tube. The supernatant supplemented with 1 μ Slit2 primary antibody was placed on a rotating shaker overnight at 4°C. The supernatant was discarded and the precipitation was washed with cold PBS for 5 times.

## RNA interference

The OLFML3-knockdown siRNA was constructed by GenePharma Co., Ltd. (Shanghai, China). The cells were transfected with siRNA using lipofectamine 3000 (Invitrogen, USA) according to the manufacturer’s instruction. The oligonucleotides sequences were listed in below.

si-OLFML3:

sense 5’-GCUACCAGAUUGUCUAUAATT-3’

antisense 5’-UUAUAGACAAUCUGGUAGCTT-3’

## Antibodies

ROBO1:

IHC, WB (Affinity Cat# DF9877, RRID: AB_2843071)

IF, WB (Thermo Fisher Scientific Cat# PA5-99084, RRID: AB_2813697)

ROBO1^E280*^:

IF, WB (Thermo Fisher Scientific Cat# PA5-99084, RRID: AB_2813697)

SLIT2:

IP, WB (GeneTex Cat# GTX118220, RRID: AB_10618954)

OLFML3:

WB (GeneTex Cat# GTX119826, RRID: AB_10617595)

VEGF:

IHC, WB (Proteintech Cat# 19003-1-AP, RRID: AB_2212657)

MMP2:

WB (Proteintech Cat# 10373-2-AP, RRID: AB_2250823),

Cyclin D1:

WB (Proteintech Cat# 26939-1-AP, RRID: AB_2880691)

PCNA:

WB (Proteintech Cat# 10205-2-AP, RRID: AB_2160330)

GAPDH:

WB (Abcam Cat# ab181602, RRID: AB_2630358)

Ki67:

IHC (Abcam Cat# ab15580, RRID: AB_443209)

CD31:

IHC (Abcam Cat# ab9498, RRID: AB_307284)

# Supplementary figures


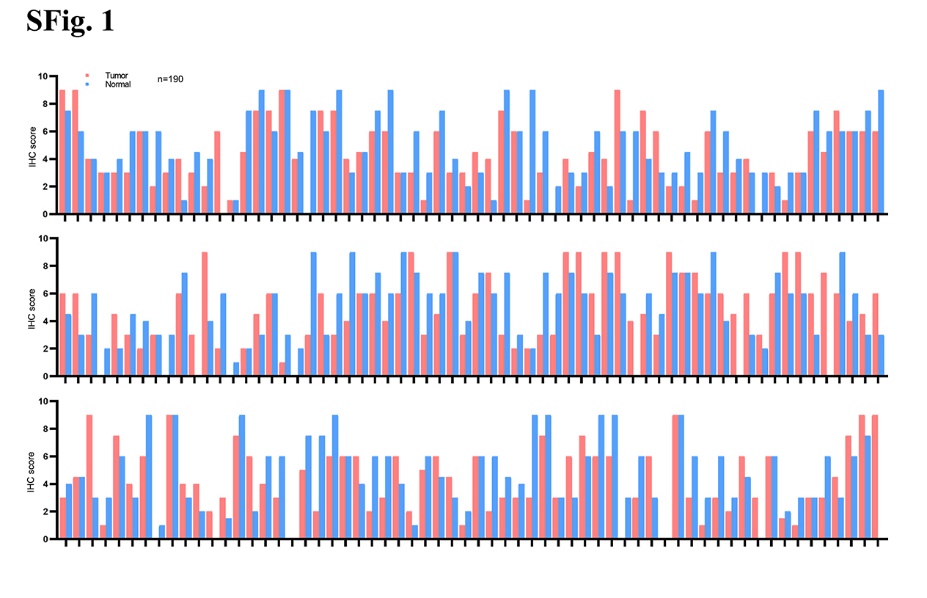


**Supplementary Figure. 1 IHC scores of tissue microarrays. (a)** Detailed IHC scores of ROBO1 expression in tissue microarrays containing 190 pairs of CCA tissues and matched normal tissues.


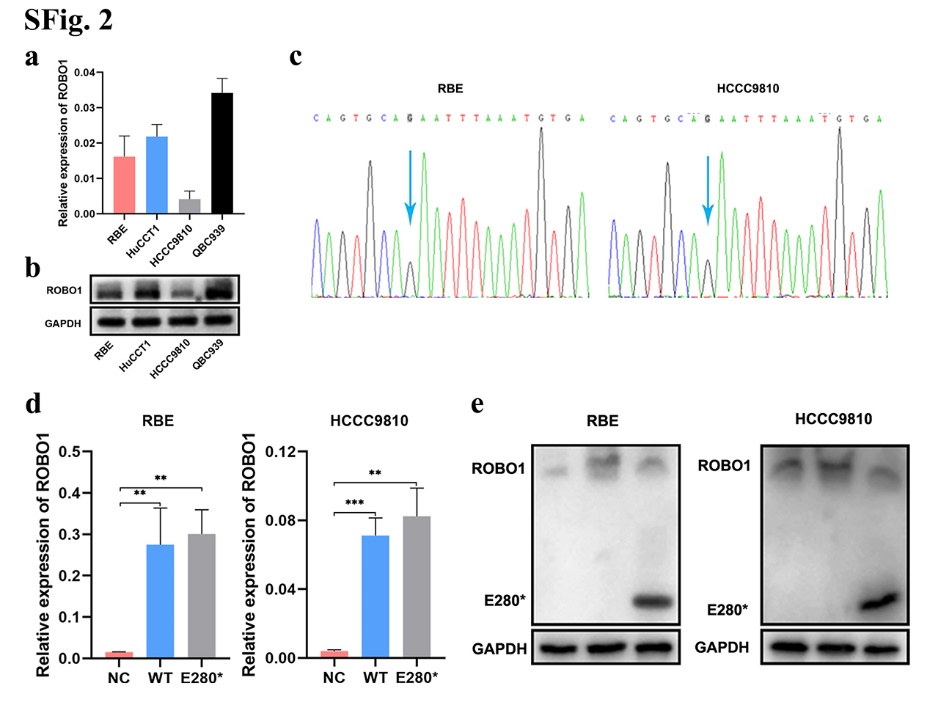


**Supplementary Figure. 2 Construction of stable LV-ROBO1^WT^ and LV-ROBO1^E280*^ cells. (a, b)** RT-qPCR and western blotting analysis of ROBO1 expression in RBE, HuCCT1, HCCC9810, and QBC939 cell lines. **(c)** Sanger sequencing of RBE and HCCC9810 to detects ROBO1 mutation. **(d, e)** Stable overexpression of ROBO1^WT^ and ROBO1^E280*^ in RBE and HCCC9810 cells. RT-qPCR analysis was used to verify the overexpression efficiency and western blotting results confirmed the existence of the truncated protein encoded by ROBO1^E280*^. **P < 0.05, **P < 0.01, ***P < 0.001.*


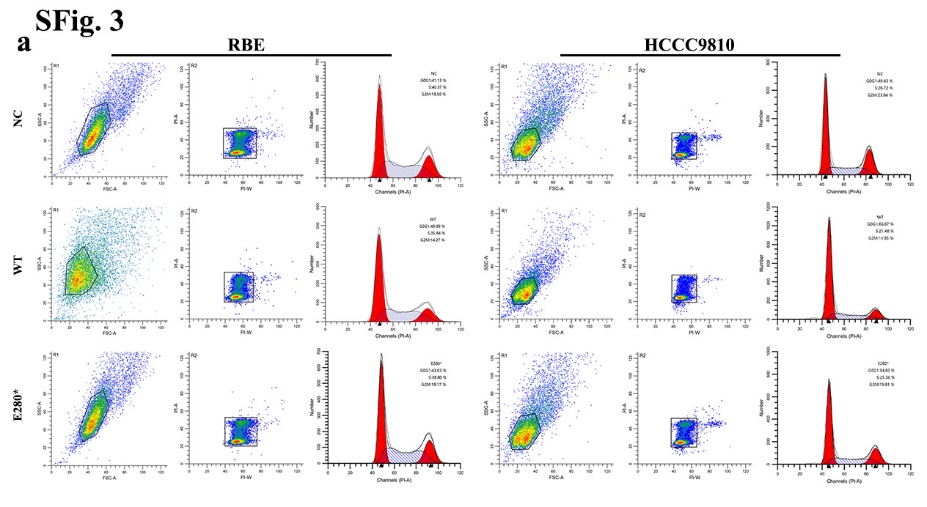


**Supplementary Figure. 3 ROBO1^E280*^ repressed the tumor-suppressing effects of ROBO1^WT^ on proliferation in CCA cells. (a)** Supplementary scatter diagrams for Fig. 2c. Flow cytometry analyzed cell cycle of CCA cells transfected with LV-ROBO1^WT^, LV-ROBO1^E280*^, and LV-NC.


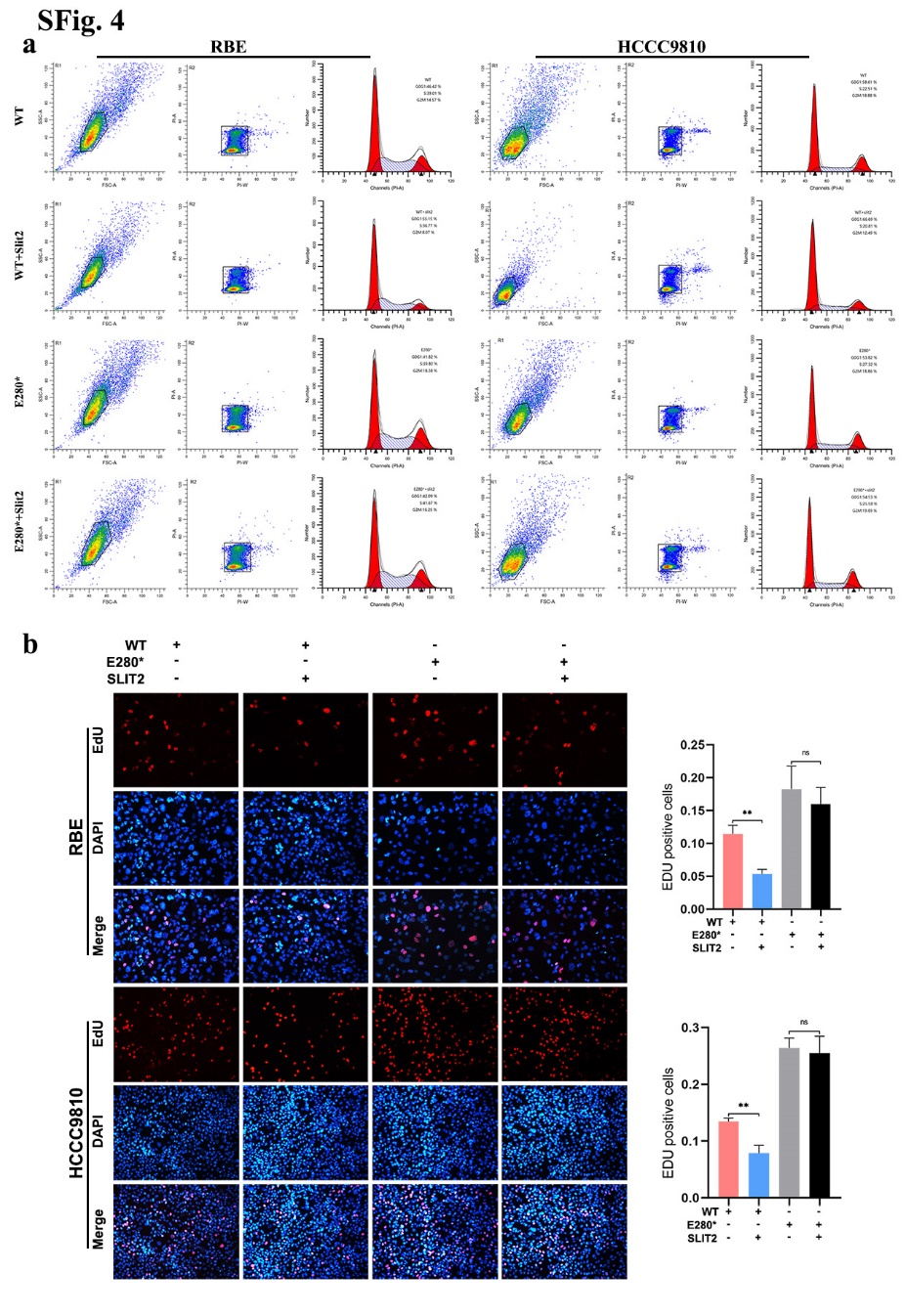


**Supplementary Figure. 4 ROBO1^E280*^ disrupted SLIT2-induced tumor-suppressing effects on proliferation and angiogenesis in CCA. (a)** Supplementary scatter diagrams for Fig. 5c. Flow cytometry analyzed cell cycle of wild-type and mutated RBE cells treated by SLIT2. **(b)** The number of EdU positive RBE cells was decreased by SLIT2 treatment in ROBO1^WT^ cells but was not in ROBO1^E280*^ cells. ***P < 0.01, ns P > 0.05.*


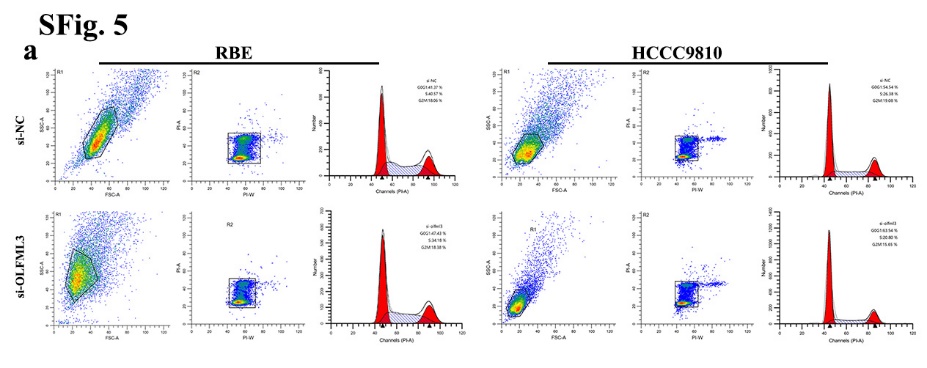


**Supplementary Figure. 5 OLFML3 was a potential target regulated by ROBO1^WT^ and ROBO1^E280*^. (a)** Supplementary scatter diagrams for Fig. 6j. Flow cytometry analyzed the cell cycle of RBE and HCCC9810 cells transfected with si-OLFML3 and si-NC.


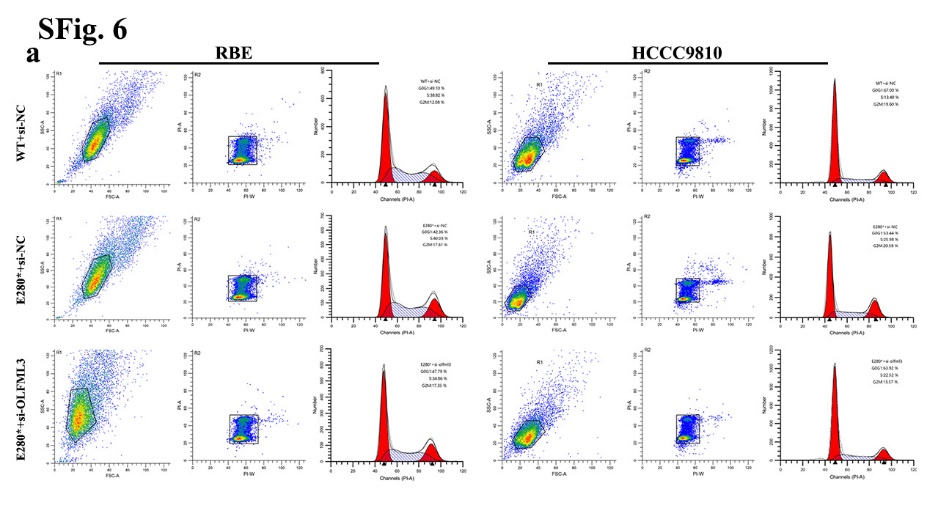


**Supplementary Figure. 6 OLFML3 was essential for ROBO1^E280*^-induced proliferation and angiogenesis in CCA. (a)** Supplementary scatter diagrams for Fig. 7c. Flow cytometry analyzed the cell cycle of ROBO1^WT^ cells transfected with si-NC, ROBO1^E280*^ cells transfected with si-NC, and ROBO1^E280*^ cells transfected with si-OLFML3.
